# MicroRNA-497 inhibits tumor growth and increases chemosensitivity to 5-fluorouracil treatment by targeting KSR1

**DOI:** 10.18632/oncotarget.6545

**Published:** 2015-12-09

**Authors:** Lin Wang, Cheng-fei Jiang, Dong-mei Li, Xin Ge, Zhu-mei Shi, Chong-yong Li, Xue Liu, Yu Yin, Linlin Zhen, Ling-Zhi Liu, Bing-Hua Jiang

**Affiliations:** ^1^ State Key Lab of Reproductive Medicine, Collaborative Innovation Center for Cancer Personalized Medicine, Department of Pathology, Nanjing Medical University, Nanjing, China; ^2^ Department of Breast and Thyroid Surgery, Huai'an First People's Hospital, Huai'an, Jiangsu, China; ^3^ Ninggao Personalized Medicine and Technology Innovation Center, Nanjing, China; ^4^ Department of Neurosurgery, The First Affiliated Hospital of Nanjing Medical University, Nanjing, China; ^5^ Department of Pathology, Anhui Medical University, Hefei, China; ^6^ Department of Pathology, Anatomy and Cell Biology, Thomas Jefferson University, Philadelphia, PA, USA

**Keywords:** miR-497, colorectal cancer, KSR1, tumorigenesis, chemosensitivity

## Abstract

Colorectal cancer (CRC) is one of the leading cancer-related causes of death in the world. Recently, downregulation of microRNA-497 (miR-497) has been observed in CRC tissues. In this study, we found that miR-497 expression levels were downregulated in human CRC specimens compared to the adjacent normal tissues. MiR-497 expression levels were strongly correlated with clinical stages and lymph node metastases. Furthermore, kinase suppressor of ras 1 (KSR1), a known oncogene, was a direct target of miR-497, and KSR1 expression levels were inversely correlated with miR-497 expression levels in human CRC specimens. Overexpression of miR-497 inhibited cell proliferation, migration, invasion and increased chemosensitivity to 5-fluorouracil treatment, whereas forced expression of KSR1 had the opposite effect. Taken together, these results revealed that lower miR-497 levels in human CRC tissues induce KSR1 expression which is associated with CRC cancer occurrence, advanced stages, metastasis and chemoresistance. Lower miR-497 levels may be a potential biomarker for CRC advanced stages and treatment response.

## INTRODUCTION

Colorectal cancer (CRC) is one of the most prevalent carcinomas throughout the world, with an estimated one million new cases and half million mortalities each year [1, 2]. The 5-year overall survival rate ranges from 40% to 60% [3, 4]. The advances of diagnostic and therapeutic approaches have greatly improved long-term survival of CRC, but a significant proportion of patients still develop into drug resistance, relapse and poor outcomes [5–7]. At the molecular level, colorectal cancer arises from a series of genetic and epigenetic alterations that may inactivate tumor suppressor genes or/and activate oncogenes [8]. However, the basic mechanisms underlying CRC initiation and progression remain largely unknown. Better understanding of the molecular mechanisms underlying carcinogenesis, progression and drug resistance in CRC would be helpful in improving diagnosis, therapy and prevention.

MicroRNAs (miRNAs) are endogenous small non-coding RNAs that act as negative regulators for mRNA expression via sequence-complementary targeting of the 3′-untranslated and other regions (3′-UTRs) to repress translation or mediate mRNA degradation [9]. Due to their abundance and divergence of targeting specificity, it is believed that one single miRNA can interact with multiple mRNA targets to achieve regulatory control over virtually many biological processes [10, 11]. A variety of studies have reported that miRNAs play critical roles in cell growth, differentiation, apoptosis and tumorigenesis and they can potentially act as both oncogenes and tumor-suppressor genes in a variety of tumors including colorectal cancer [12, 13]. Among them, miR-497 has been demonstrated to function as a tumor suppressor. Loss of miR-497 expression has been reported in many cancer types, whereas restoration of miR-497 expression has been shown to abrogate tumorigenesis [14–16]. To date, some genes have been identified as miR-497 target genes, including IGF-IR, eIF4E, SMURF1, Bcl2 and cyclin E1 [14, 17–20], which are involved in pathogenesis of cancers. However, the role and molecular mechanism of downregulated miR-497 in CRC has not been fully determined.

In the present study, we demonstrated that miR-497 levels were downregulated in human CRC tumor specimens using 62 pairs of normal and cancer tissues. Then, we will investigate: (1) what is the role of miR-497 in CRC cell growth, migration and invasion; (2) what is specific direct target of miR-497 that is associated with cancer development; and (3) whether forced miR-497 expression inhibits cell growth, migration and invasion *via* this direct target; (4) whether miR-497 and its target are responsible for the resistance to 5-fluorouracil treatment in CRC. These results will provide new insights into the molecular mechanism of CRC development and provide potential new therapeutic strategy for CRC treatment in the future.

## RESULTS

### MiR-497 is down-regulated in human CRC specimens

**Figure 1:**
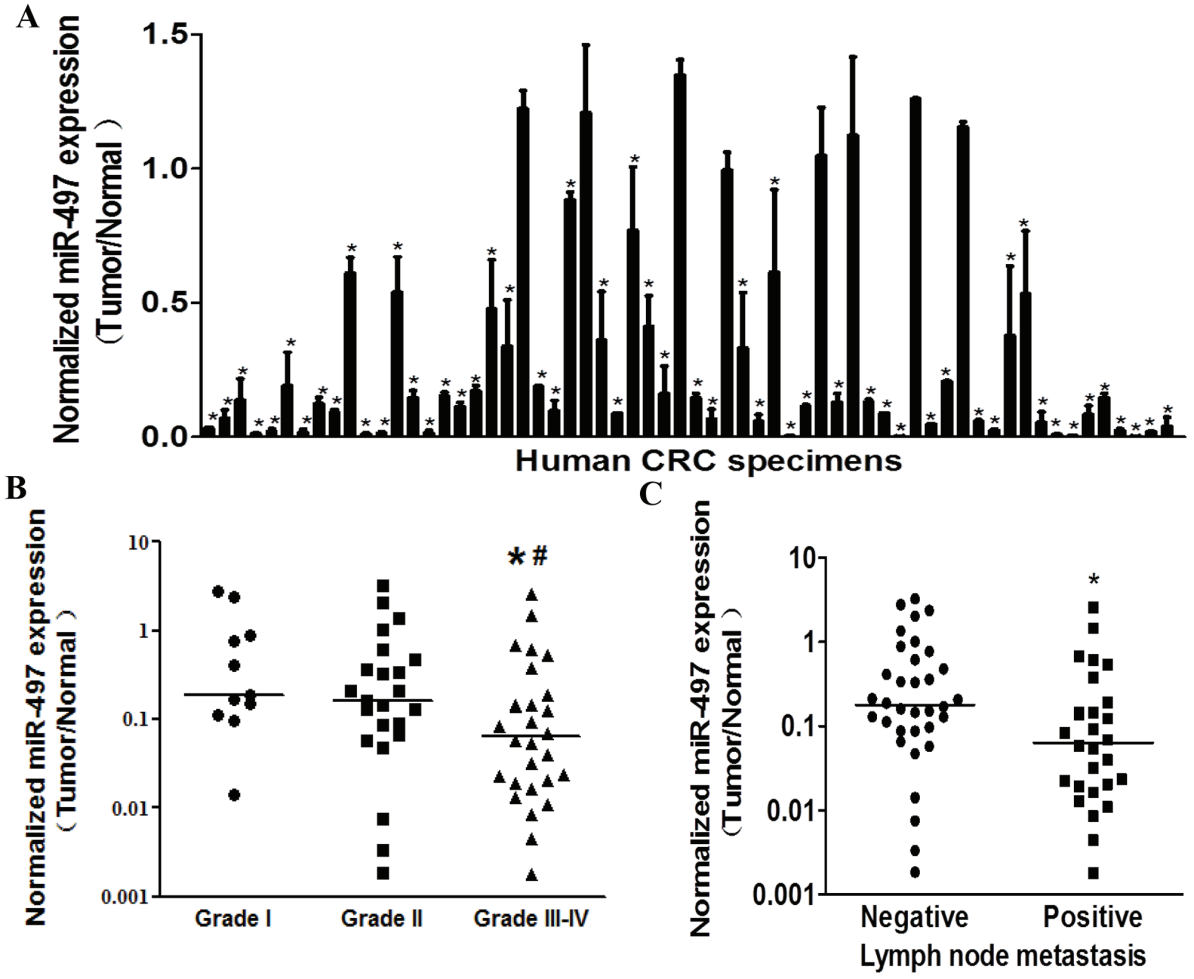
MiR-497 levels are down-regulated in human colorectal cancer tissues **A.** Relative miR-497 expression levels were analyzed by Taqman RT-qPCR in 62 pairs of CRC specimens. U6 RNA levels were used as an internal control. **B.** Relative expression levels of miR-497 in different stages of cancer tissues, * indicates significant difference at *p* < 0.05 when compared to miR-497 expression levels in Grades III-IV with those of Grades I; ^#^ indicates significant difference at *p* < 0.05 when compared miR-497 expression levels in Grades III-IV with those of Grades II. **C.** Relative expression levels of miR-497 in different types of lymph node metastasis. * indicates significant difference at *p* < 0.05 when compared miR-497 expression levels in positive lymph node metastasis with those in negative ones.

To determine the expression levels of miR-497 in human CRC specimens, RT-qPCR analysis was performed in 62 pairs of tumor specimens and matched adjacent normal tissues. The results showed that miR-497 expression levels in tumor tissues were significantly lower than those in adjacent normal ones (Figure 1A). To compare miR-497 expression levels among different clinical stages, we found that its expression levels in tumor tissues were correlated with the clinical stages of CRC patients. The expression levels of miR-497 in high grade tumors (WHO Grades III-IV) were significantly downregulated compared with those in low grade tumors (WHO Grade I and II) (Figure 1B and Table 1). In addition, miR-497 levels were markedly lower in the patients with lymph node metastases than those in the patients without lymph node metastases (Figure 1C and Table 1), which was consistent with the above result, since lymph node metastases commonly occurs in high grade tumors. Taken together, low expression levels of miR-497 in tumor tissues were closely related with advanced clinical stages and metastases, indicating that miR-497 levels may be a potential new biomarker for the diagnosis of CRC.

**Table 1:** Comparison of clinical patothologic factors and normalized expression of miR-497 in 62 pairs of CRC

Colorectal cancer	*n*	Normalized expression of miR-497^a^
Lymph node		
Negative	36	0.1786 (0.0878-0.6524)
Positive	26	0.0613 (0.0194-0.1787)
*P* value		0.0141
TNM stage		
Grade I	12	0.1872 (0.1126-0.8827)
Grade II	24	0.1607 (0.0657-0.6135)
Grade III-IV	26	0.0613 (0.0194-0.1787)
*P* value		0.0345

a Median of normalized expression of miR-497, with 25th-75th precentile in parenthesis

### MiR-497 inhibits cell proliferation, migration and invasion of CRC cells

**Figure 2:**
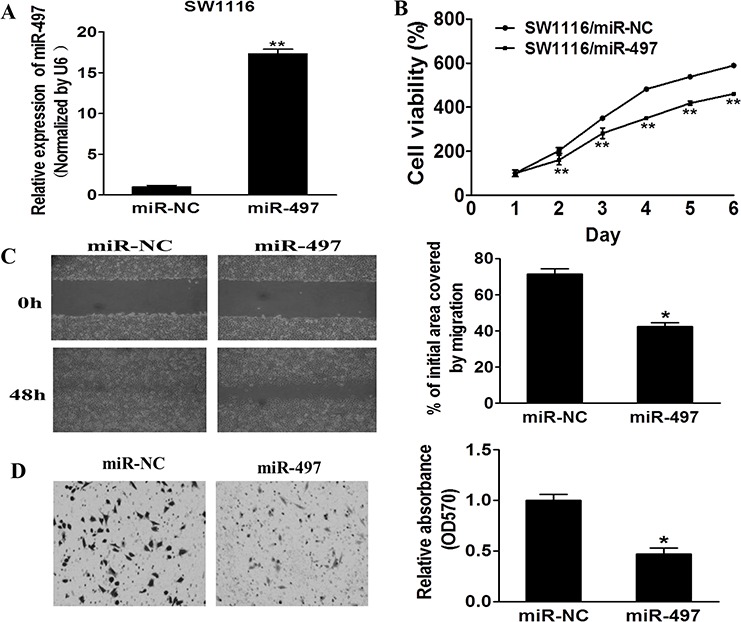
MiR-497 inhibits cell proliferation, migration and invasion **A.** Relative expression levels of miR-497 in SW1116/miR-497 and SW1116/miR-NC stable cell lines were confirmed by Taqman RT-qPCR. **B.** SW1116/miR-497 and SW1116/miR-NC cells were plated 2000 cells per well in 96-well plates, and cell proliferation was determined using Cell Counting Kit-8 (CCK-8) to detect the absorbance at 450 nm every day. **C.** SW1116/miR-497 and SW1116/miR-NC cells were cultured to 90% confluence. A sterile 200 μl pipette tip was used to scratch the cells to form a wound. The wound gaps were photographed and measured. **D.** Matrigel invasion assay of SW1116/miR-497 and SW1116/miR-NC cells were performed as previously described. Data represent mean ± SD from 3 replicates. * indicates significant difference at *p* < 0.05 when compared to the miR-NC control; ** indicates significant difference at *p* < 0.01 when compared to the miR-NC control.

To examine the role of miR-497 during carcinogenesis of human CRC, SW1116 cells with low expression levels of miR-497 were infected with lentivirus expressing miR-497 or negative control. After the selection by puromycin, stable cell lines termed as SW1116/miR-497 and SW1116/miR-NC were established. Taqman RT-PCR analysis demonstrated miR-497 was highly expressed in SW1116/miR-497 cells, confirming that stable cell line over-expressing miR-497 was successfully established (Figure 2A).

To further study the role of miRNA-497 in regulating cell proliferation, migration and invasion, we found that cell growth and migration were attenuated in SW1116/miR-497 cells compared with SW1116/miR-NC cells (Figures 2B and 2C). Furthermore, SW1116/miR-497 cells showed significantly lower invasion activity compared to SW1116/miR-NC (Figure 2D). Thus, our results show that miR-497 is responsible for suppressing cell proliferation, migration and invasion, similarly as a tumor suppressor in CRC cells.

### KSR1 is a direct target of miR-497, and CRC tissues have higher KSR1 levels that are inversely correlated with miR-497 expression levels

**Figure 3:**
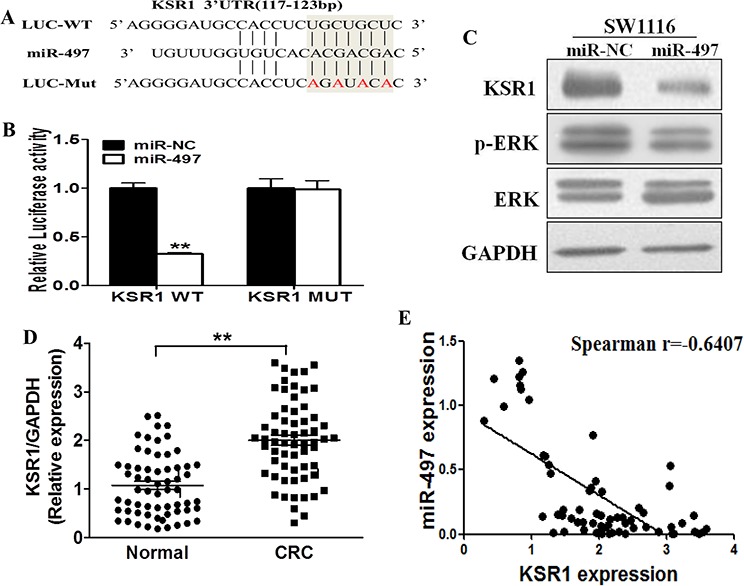
KSR1 is a direct target of miR-497, and is elevated in CRC tissues, which is inversely correlated with miR-497 expression levels **A.** The complementary pairings of miR-497 with KSR1 wild-type (WT) and mutant (MUT) 3′ UTR reporter constructs are shown. The mutant nucleotides of the KSR1 3′-UTR are labeled in red. **B.** The reporter constructs containing the WT or MUT KSR1 3′ UTR regions were co-transfected with miR-NC or miR-497 and pRL-TK plasmids into SW1116 cells. The luciferase activities were analyzed in the cells 24 h after the transfection. The data are means ± SE from separate experiments (*n* = 4). ** indicates significant difference at *p* < 0.01 when compared to the miR-NC control. **C.** SW1116 cells were transfected with miR-497 or miR-NC plasmids as above. After 72 h culture, expression levels of KSR1, p-ERK, ERK and GAPDH in the cells were determined using immunoblotting assay. **D.** The expression levels of KSR1 in normal tissues and human CRC specimens were determined by Western blotting analysis, the density signals were quantified using ImageJ software, and fold changes were obtained by the ratios of KSR1 to GAPDH levels. ** indicates significant difference at *p* < 0.01 when compared to those of adjacent normal tissues. **E.** Spearman's correlation analysis was used to determine the correlations between the expression levels of KSR1 and miR-497 in human CRC specimens.

To fully understand mechanism of miR-497 in inhibiting human CRC development, TargetScan search program was used to predict targets of miR-497. KSR1 was one of the putative targets of miR-497 (Figure 3A). To explore whether miR-497 targets KSR1 by binding to its 3′-UTR region, SW1116 cells were co-transfected with the wild type (WT) or mutant (Mut) KSR1 luciferase reporter in the presence of miR-497 or miR-NC. After 24 h, the luciferase activities in these cells were measured. As shown in Figure 3B, luciferase activities were significantly reduced in the cells transfected with the wild type KSR1 reporter, but not in the cells with the mutant reporter (Figure 3B). In addition, forced expression of miR-497 attenuated KSR1 protein expression and ERK activation, a known downstream molecule (Figure 3C), suggesting that miR-497 directly targets KSR1 by binding its seed region of the 3′-UTR region in human CRC cells.

Furthermore, we measured levels of KSR1 in human CRC specimens and adjacent normal tissues. The results showed that the average expression levels of KSR1 were significantly higher in tumor tissues than those in the normal tissues (Figure 3D). Next, we determine the correlation between KSR1 levels and miR-497 expression levels in the same human CRC specimens using Spearman's rank correlation analysis. As shown in Figure 3E, expression levels of KSR1 and miR-497 were inversely correlated in 62 human CRC specimens (Spearman's correlation r=-0.6407).

### Restoration of KSR1 reverses miR-497-suppressed cell proliferation, migration and invasion

**Figure 4:**
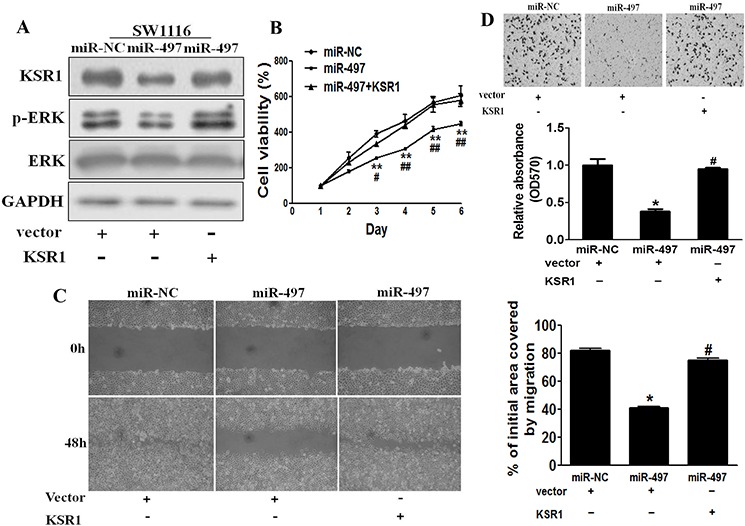
Overexpression of KSR1 reverses miR-497-mediated suppression of cell proliferation, migration and invasion **A.** SW1116 cells were co-tranfected with miR-497 or miR-NC, and with empty vector or KSR1 cDNA without 3′-UTR region. After 72 h culture, immunoblotting assay was performed as described above. **B.** Cells were treated as above, and cell proliferation assay was performed using CCK-8 kit. **C.** Cells were transfected as described above. A sterile 200 μl pipette tip was used to scratch the cells to form a wound. The wound gaps were photographed and measured. **D.** miR-497 overexpression decreased invasion of SW1116 cells, and overexpression of KSR1 reverses the inhibitory effects of miR-497. The cells treated as above were subjected to a Matrigel invasion assay. Data represent mean ± SD of three replicates. * indicates significant difference at *p* < 0.05 compared to miR-NC control; ** indicates significant difference at *p* < 0.01 compared to miR-NC control. # indicates significant difference at *p* < 0.05 compared to miR-497 and KSR1 overexpression; ^##^ indicates significant difference at *p* < 0.01 compared to miR-497 and KSR1 overexpression.

To test whether KSR1 overexpression reverses miR-497-inhibiting cell proliferation, migration and invasion, SW1116 cells were transfected with KSR1 cDNA without its 3′-UTR region. As shown in Figure 4A, forced expression of KSR1 rescued miR-497-suppressed KSR1 expression and ERK activation, confirming the effect of KSR1 cDNA expression. As we expected, forced expression of KSR1 also restored miR-497-inhibited cell proliferation, migration and invasion (Figures 4B–4D), suggesting that miR-497 suppresses human CRC cell proliferation, migration and invasion by inhibiting its target KSR1.

### MiR-497 renders CRC cells more sensitive to 5-fluorouracil treatment by targeting KSR1

**Figure 5:**
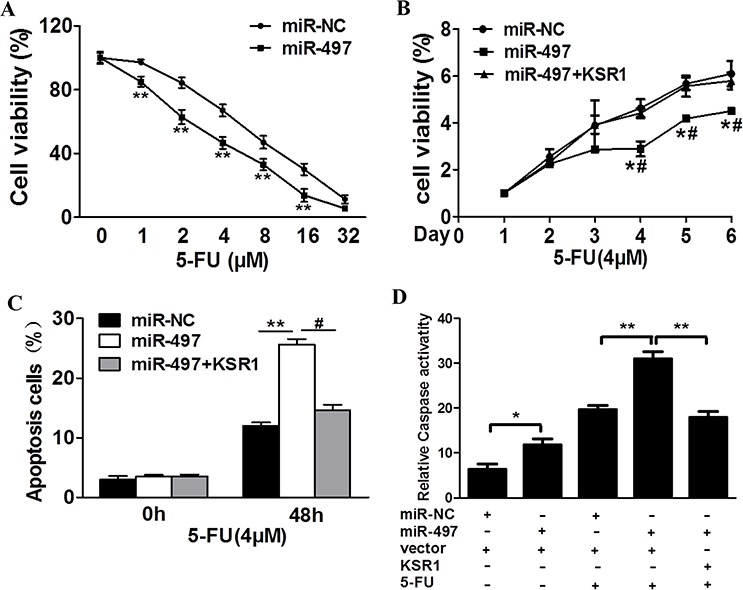
MiR-497 regulates 5-fluorouracil chemosensitivity by targeting KSR1 in colorectal cancer cells **A.** SW1116 cells stably expressing miR-NC or miR-497 were treated with different concentrations of 5-fluorouracil for 48 h, and analyzed by CCK-8 Assay. **B.** SW1116 cells stably expressing miR-NC, miR-497, or miR-497 in combination with KSR1 overexpression were treated with 4 μM of 5-fluorouracil for indicated time points. Cell proliferation was analyzed by CCK-8 Assay, and cell apoptosis was analyzed by flow cytometry **C.** and by caspase-3 assay **D.** Data represent mean ± SD from three replicates. * indicates significant difference at *p* < 0.05 compared to miR-NC control; ** indicates significant difference at *p* < 0.01 compared to miR-NC control. ^#^ indicates significant difference at *p* < 0.05 compared to miR-497 and KSR1 overexpression. 5-FU, 5-fluorouracil.

Resistance to 5-fluorouracil treatment is one of the major causes for the failure of chemotherapy in treating human CRC. Therefore, it is critical to discover new strategies to increase 5-fluorouracil effect for therapeutic purposes. Our results showed that overexpression of miR-497 in SW1116 cells significantly increased chemosensitivity to 5-fluorouracil treatment (Figure 5A). Furthermore, cell growth rate in the presence of 5-fluorouracil was assayed using CCK-8 proliferation assay at different time points, we found that forced expression of KSR1 resulted in more resistance to 5-fluorouracil treatment in miR-497-overexpressing CRC cells (Figure 5B). To further test whether miR-497 and its target KSR1 play a role in cellular apoptosis in the presence of 5-fluorouracil treatment, FACS analysis and caspase-3 assay were performed. The combination of miR-497 and 5-fluorouracil treatment significantly induced cellular apoptosis, whereas forced expression of KSR1 partially abolished the apoptotic effect induced by the combination of miR-497 and 5-fluorouracil treatments (Figure 5C). Moreover, we found that compared with miR-497 or 5-fluorouracil treatment alone, the activities of caspase-3, a key executor of cell apoptosis, were significantly upregulated upon combination treatment of miR-497 and 5-fluorouracil, whereas forced expression of KSR1 attenuated the activation of caspase-3 during the treatment (Figure 5D). These results indicate that miR-497 renders CRC cells more sensitive to 5-fluorouracil treatment for inducing apoptosis through targeting KSR1.

### MiR-497 suppresses tumorigenesis *in vivo*

**Figure 6:**
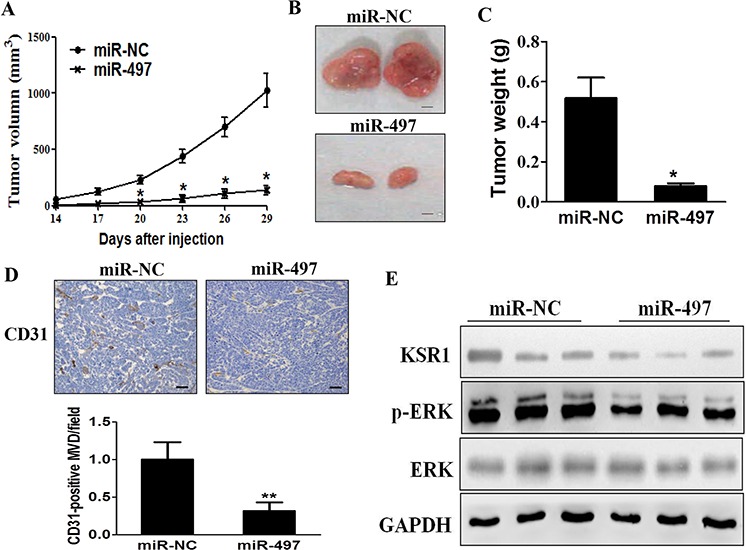
MiR-497 overexpression suppresses tumorigenesis and decreases KSR1 expression in tumor tissues **A.** SW1116/miR-497 or SW1116/miR-NC cells (5 × 10^6^ cells) were dispersed in 100 μL (l of serum-free RPMI 1640 medium, and subcutaneously injected into both sides of posterior flanks of the nude mice (*n* = 8). Tumors were measured every three days after they were apparently detectable at Day 14. Tumor volumes were calculated using the following formula: volume = 0.5 × (length × width^2^). **B.** SW1116/miR-497 cells produced smaller tumors than control 30 days after injection. Representative images of tumors were shown (Bar = 2 mm). **C.** Average weights of tumors from SW1116/miR-497 and SW1116/miR-NC groups. MiR-497 overexpression showed lower tumor weight compared with miR-NC. **D.** The expression levels of CD31 were analyzed in tumor tissues using immunohistochemistry. The representative images are shown. The CD31 levels in tissues were quantified by ImageJ software. Magnification, × 200, Scale bar, 50 μm. **E.** The total proteins were extracted from xenografts and subjected to immunoblotting assay to test levels of KSR1 and other proteins as indicated. GAPDH levels were used as the internal control. Data were presented mean ± SD (*n* = 8). * indicates significant difference at *p* < 0.05 compared to miR-NC group; ** indicates significant difference at *p* < 0.01 compared to miR-NC group.

In order to test whether miR-497 inhibits tumor growth and angiogenesis of CRC *in vivo*, SW1116/miR-497 or SW1116/miR-NC cells were injected into both posterior flanks of immunodeficient mice, and tumor sizes were started to be measured after 2 weeks of injection. Compared to miR-497 group, miR-NC group developed significantly larger tumors from Day 20 to Day 29 (Figure 6A). MiR-497 overexpression significantly suppressed tumor growth since the tumors trimmed out from miR-497 overexpressing group showed smaller size and lower tumor weight (Figures 6B and 6C). Immunohistochemistry (IHC) staining revealed that expression levels of CD31 and the quantitative microvascular density (MVD) were significantly decreased by miR-497 overexpression in tumor tissues (Figure 6D), demonstrating that miR-497 inhibits angiogenesis in xenografts. Consistent with *in vitro* data, levels of KSR1 and p-ERK1/2 in tumor tissues from miR-497 overexpression group were much lower than those of miR-NC group analyzed by immunoblotting assay (Figure 6E). Taken together, these results suggest that miR-497 inhibits tumor growth and angiogenesis with decreased KSR1 expression *in vivo*.

## DISCUSSION

MicroRNAs (miRNAs), a novel class of regulatory molecules, have been frequently indicated to be dysregulated in diverse human cancers [21]. miRNAs have been documented to function as both tumor suppressor genes and oncogenes in regulating many cellular events. Recent studies have reported that miR-497 functions as a tumor suppressor in several kinds of cancers [14–20]. In this study, we found that miR-497 expression was downregulated in CRC tumor samples compared with adjacent normal tissues. Overexpression of miR-497 inhibited cell proliferation, migration, invasion and increased chemosensitivity to 5-fluorouracil treatment.

KSR1 is a molecular scaffold for the Raf/MEK/ERK phosphorylation cascade [22–24]. KSR1 binds to Raf, MEK, and ERK to positively induce ERK activation [22–27]. KSR1 has been widely denoted as a pseudokinase, owing to the fact that there is controversy regarding whether its kinase domain is active. However, emerging evidence suggests that KSR1 has dual functions as both an active kinase and a scaffolding protein, adding complexity to the simple view of MAPKs spatiotemporal pathway control [28, 29]. Recent studies have reported that KSR1 affects cell proliferation and oncogenic transformation through inducing ERK activation [22]. In our study, KSR1 oncogene has been experimentally validated as a novel direct target of miR-497 not only *in vitro*, but also *in vivo*. Furthermore, we also show that miR-497 acts as a tumor suppressor to inhibit cell growth, migration, invasion, and to diminish MAPK/ERK signaling pathway *via* targeting KSR. Our study highlights a key role of miR-497 in inhibiting CRC tumorigenesis.

Fluoropyrimidine-based chemotherapy (e.g., 5-fluorouracil (5-FU), S-1) has been the cornerstone of treating advanced CRC for over a half century. Extensive efforts in the past have contributed to better understanding of both molecular and cellular mechanisms of 5-fluorouracil action, one of the most important pyrimidine antagonists [30]. Recent studies have focused on the involvement of microRNA (miRNA) in cancer chemoresistance. miRNAs are differentially expressed in chemosensitive and chemoresistant cells [31, 32]. Several studies have suggested that miRNAs are novel players in the development of chemoresistance. Our recent study showed that microRNA-143 inhibits tumor growth and angiogenesis and sensitizes chemosensitivity to oxaliplatin in colorectal cancers [33]. Overexpression of miR-22 reverses paclitaxel-induced chemoresistance through activation of PTEN signaling in p53-mutated colon cancer cells [34]. In this study, we found that forced expression of miR-497 increased the effect of 5-fluorouracil in inducing cellular apoptosis, indicating that miR-497 restoration may offer a new option to overcome chemoresistance to 5-fluorouracil treatment in CRC.

In summary, we have identified that KSR1 is a novel target of miR-497. MiR-497 acts as a tumor suppressor to inhibit cell proliferation, migration, invasion, tumor growth and angiogenesis *via* targeting KSR1. In addition, overexpression of miR-497 renders CRC cells more sensitive to 5-fluorouracil treatment, suggesting that miR-497 may be used as a combination therapy for CRC treatment in the future.

## MATERIALS AND METHODS

### Clinical specimens

Human CRC specimens (62 pairs) and adjacent normal tissues were obtained from Nanjing Medical University and Anhui Medical University, China. All tissue samples were snap-frozen in liquid nitrogen immediately after surgery and stored in liquid nitrogen. All samples were histologically classified by clinical pathologist. Samples used for molecular analysis were initially grinded into powder in liquid nitrogen and then collected separately for RNA or protein analyses. The experiment protocols have been approved by the ethics committees of Nanjing Medical University, Nanjing, China; and Anhui Medical University, Hefei, China.

### Cell culture and reagents

Human CRC cells SW1116 were cultured in RPMI 1640 medium, and HEK293T cells were cultured in DMEM medium supplemented with 10% fetal bovine serum, 100 units of penicillin/ml and 100 ng of streptomycin/ml. Cells were incubated at 37°C in a humidified chamber supplemented with 5% CO_2_. Antibodies against KSR1, p-ERK1/2 and ERK1/2 were purchased from Cell Signaling Technology (Danvers, MA, USA). Antibody against GAPDH was from Bioworld Technology (Atlanta, Georgia 30305, USA). The growth factor reduced Matrigel was from BD Biosciences (Bedford, MA, USA).

### Lentiviral packaging and stable cell line establishment

To stably overexpress miR-497 in CRC cells, the lentiviral packaging kit was used (Thermo Fisher Scientific). Lentivirus carrying miR-497 or negative control (miR-NC) was packaged using HEK293T cells following the manufacturer's manual. The lentiviral vector has red fluorescent protein (RFP) tag which can be used to check the efficiency of packaging using microscope. SW1116 cells were infected by lentivirus carrying miR-497 or miR-NC in the presence of polybrene (Sigma-Aldrich) and selected by puromycin (Sigma-Aldrich) for two weeks to obtain SW1116/miR-497 and SW1116/miR-NC stable cell lines.

### Total RNA extraction, reverse transcription PCR and quantitative real time-PCR

Total RNAs were isolated from harvested cells or human tissues using Trizol reagent according to the manufacturer's instruction (Invitrogen, CA, USA). To measure expression levels of miR-497, Taqman real-time PCR assay was performed and the U6 was used as an endogenous control. To determine the mRNA levels of KSR1, total RNAs were reversely transcribed by oligodT primer using RT Reagent Kit (Vazyme, Nanjing, China). Housekeeping gene GAPDH was used as internal control. The cDNAs were amplified by real-time PCR using SYBR Green Master Mix (Vazyme, Nanjing, China) on a 7900HT system, and fold changes were calculated by relative quantification (2^−△△Ct^). Primers were listed in Supplementary Table S1.

### Immunoblotting

Cells were washed with ice-cold PBS buffer, scraped from the dishes, and centrifuged at 12,000 rpm, 4°C for 15 min. Cell lysates were prepared using RIPA buffer supplemented with protease inhibitors (100 mM Tris, pH 7.4, 150 mM NaCl, 5 mM EDTA, 1% Triton X-100, 1% deoxycholate acid, 0.1% SDS, 2 mM phenylmethylsulfonyl fluoride, 1 mM sodium orthovanadate, 2 mM DTT, 2 mM leupeptin, 2 mM pepstatin). The supernatants were collected and protein concentrations were determined using BCA assay (Beyotime Institute of Biotechnology, Jiangsu, China). Tumor tissues from human and nude mice were grinded into powder in liquid nitrogen with RIPA buffer, and the total tissue proteins were extracted as described above. Aliquots of protein lysates were fractionated by SDS-PAGE, transferred to a PVDF membrane (Roche, Switzerland), and subjected to immunoblotting analysis according to the manufacturer's instruction. ECL Detection System (Thermo Scientific, Rockford, IL, USA) was used for protein signal detection.

### Cell proliferation assay

To evaluate the proliferation effect of miR-497 in CRC cells, SW1116/miR-497 and SW1116/miR-NC stable cell lines were plated at a density of 2 × 10^3^ cells per well in 96-well plate incubated at 37°C in 5% CO_2_ incubator. The cell proliferation was analyzed using a CCK-8 kit (Dojindo Laboratories, Kumamoto, Japan) according to the manufacturer's instruction. Data were from three separate experiments with six replications per experiment.

### Wound healing assay

Cells were cultured until 90% confluence in 6-well plates. Cell layers were scratched using a 200 μL tip to form wounded gaps, washed with 1x PBS buffer twice and cultured for different time points. The wounded gaps were photographed and analyzed by measuring the distance of migrating cells from five different areas for each wound. Three independent experiments were conducted in triplicate.

### *In vitro* invasion assay

Invasion assay was determined using 24-well BD Matrigel invasion chambers (BD Biosciences, Cowley, UK) in accordance with the manufacturer's instruction. SW1116/miR-497 and SW1116/miR-NC cells were plated at a density of 5 × 10^4^ cells per well in the upper chamber without serum. The lower chamber was filled with 600 ml of the RPMI 1640 medium with 10% FBS to act as the nutritional attraction. After incubation for 24 h, noninvading cells were removed from the top well with a cotton swab, while the bottom cells were fixed with 3% paraformaldehyde, stained with 0.1% crystal violet, and photographed in three independent fields for each well. They were finally extracted with 33% acetic acid and detected quantitatively using a standard microplate reader (OD at 570 nm). Three independent experiments were conducted in triplicate.

### Dual-luciferase reporter assay

For dual-luciferase assay, 3′-UTR regions of KSR1 containing predicted miR-497 seed-matching sites and corresponding mutant sites were amplified by PCR using human cDNA template, and inserted into the SacI and HindIII restriction enzyme sites by pMIR-REPORTER vector (Ambion, CA, USA). These constructs were validated by DNA sequencing. SW1116 cells were seeded in a 24-well plate and co-transfected with the wild type or mutant reporter plasmid, pRL-TK plasmid, and miR-497 or miR-NC. Luciferase activities in the cells were analyzed 24 h after transfection using the Dual Luciferase Reporter Assay System (Promega, WI, USA). Experiments were performed in three independent replicates.

### *In Vitro* chemosensitivity array

Cancer cells were seeded at a density of 4,000 cells per well in a 96-well plate overnight. Freshly prepared 5-fluorouracil solution (Sigma-Aldrich, St. Louis, MO, USA) was added into medium to obtain final culture concentrations ranging from 1 to 32 μM. Cell viability was assayed using CCK8 kit 48 h later.

### Apoptosis assay

Apoptosis was measured by flow cytometry as previously described [35]. For AnnexinV staining, 5 μL phycoerythrin-Annexin V, 5 μL propidium iodide (BD Pharmingen) and 400 μL 1× binding buffer were added to the cell samples, and incubated for 15 min at room temperature in the dark. Then the samples were analyzed by flow cytometry (FACSCanto II, BD Biosciences) within 1 h. The data were analyzed using FlowJo software. Three experiments were performed in triplicate.

### *In vivo* tumorgenesis assay

Male nude mice (BALB/c-null, 6-week-old) were purchased from Shanghai Laboratory Animal Center (Chinese Academy of Sciences, Shanghai, China), and bred in special pathogen-free (SPF) condition. Cells (5 × 10^6^) were suspended in 100 μL of serum-free RPMI 1640 medium, and injected subcutaneously into each side of the posterior flank of each nude mouse. Tumor sizes were measured every three days from the 14^th^ day of injection. Tumor volumes were calculated using vernier caliper using the formula: volume = 0.5 × (Length × Width^2^). Mice were sacrificed 30 days after injection, and tumors were dissected. Total proteins were extracted and used for immunoblotting. Tumors were formalin-fixed, paraffin-embedded, sectioned at 5μm, then immunohistochemically stained using CD31 antibody (Abcam, Cambridge, UK) under similar procedure as previously described [36].

### Statistical analysis

All experiments were performed three times, and data were analyzed with GraphPad Prism 5(La Jolla, CA, USA). The correlations between miR-497 expression levels and clinical pathologic features in human CRC specimens were analyzed by Mann-Whitney test for 2 groups and by Kruskall-Wallis test for 3 groups. The correlations between miR-497 expression levels and KSR1 levels in human CRC tissues were analyzed using Spearman's rank test. Statistical evaluation for data analysis was determined by *t*-test. The differences were considered to be statistically significant at *P* < 0.05.

## SUPPLEMENTARY TABLE


