# Supplementary material for: MicroRNA-497 inhibits tumor growth and increases chemosensitivity to 5-fluorouracil treatment by targeting KSR1
Source: Oncotarget. 2015 Dec 9;7(3):2660–71. doi: 10.18632/oncotarget.6545 (PMC4823062; doi:10.18632/oncotarget.6545)
Supplement: Supplementary file 1 [file oncotarget-07-2660-s001.pdf]

SUPPLEMENTARY TABLE

Supplementary Table S1: Primers Used in This Study

Primer name	Sequence 5′-3′
miR-497 RT	CTCAACTGGTGTCGTGGAGTCGGCAATTCAGTTGAGAACAAACC
miR-497-F	ACACTCCAGCTGGGCAGCAGCACACTGTGG
miR-497-R	TGGTGTCGTGGAGTCG
U6 RT	AACGCTTCACGAATTTGCGT
U6-F	CTCGCTTCGGCAGCACA
U6-R	TGGTGTCGTGGAGTCG
WT-KSR-F	CCAAGCTTCACCGTTGCTGCTCCAAGTAGG
WT-KSR-R	GACTAGTCGTCGTGGTGGTTGTTGGGAT
MT-KSR-F	CCAAGCTTCACCGTAGATACACCAAGTAGG
